# Increased Dendritic Branching of and Reduced δ-GABA_A_ Receptor Expression on Parvalbumin-Positive Interneurons Increase Inhibitory Currents and Reduce Synaptic Plasticity at Puberty in Female Mouse CA1 Hippocampus

**DOI:** 10.3389/fncel.2020.00203

**Published:** 2020-07-09

**Authors:** Hui Shen, Lindsay Kenney, Sheryl S. Smith

**Affiliations:** ^1^Department of Physiology and Pharmacology, SUNY Downstate Medical Center, Brooklyn, NY, United States; ^2^Research Institute of Neurology, General Hospital, Tianjin Medical University, Heping District, Tianjin, China; ^3^Program in Neural and Behavioral Science, SUNY Downstate Medical Center, Brooklyn, NY, United States; ^4^The Robert F. Furchgott Center for Neural and Behavioral Science, SUNY Downstate Medical Center, Brooklyn, NY, United States

**Keywords:** parvalbumin, interneurons, CA1 hippocampus, puberty, delta, GABA_A_ receptor, dendritic branching, long-term potentiation

## Abstract

Parvalbumin positive (PV+) interneurons play a pivotal role in cognition and are known to be regulated developmentally and by ovarian hormones. The onset of puberty represents the end of a period of optimal learning when impairments in synaptic plasticity are observed in the CA1 hippocampus of female mice. Therefore, we tested whether the synaptic inhibitory current generated by PV+ interneurons is increased at puberty and contributes to these deficits in synaptic plasticity. To this end, the spontaneous inhibitory postsynaptic current (sIPSC) was recorded using whole-cell patch-clamp techniques from CA1 pyramidal cells in the hippocampal slice before (PND 28–32) and after the onset of puberty in female mice (~PND 35–44, assessed by vaginal opening). sIPSC frequency and amplitude were significantly increased at puberty, but these measures were reduced by 1 μM DAMGO [1 μM, (D-Ala^2^, N-MePhe^4^, Gly-ol)-enkephalin], which silences PV+ activity *via* μ-opioid receptor targets. At puberty, dendritic branching of PV+ interneurons in GAD67-GFP mice was increased, while expression of the δ subunit of the GABA_A_ receptor (GABAR) on these interneurons decreased. Both frequency and amplitude of sIPSCs were significantly increased in pre-pubertal mice with reduced δ expression, suggesting a possible mechanism. Theta burst induction of long-term potentiation (LTP), an *in vitro* model of learning, is impaired at puberty but was restored to optimal levels by DAMGO administration, implicating inhibition *via* PV+ interneurons as one cause. Administration of the neurosteroid/stress steroid THP (30 nM, 3α-OH, 5α-pregnan-20-one) had no effect on sIPSCs. These findings suggest that phasic inhibition generated by PV+ interneurons is increased at puberty when it contributes to impairments in synaptic plasticity. These results may have relevance for the changes in cognitive function reported during early adolescence.

## Introduction

The onset of puberty is a transition phase associated with many changes in behavior including an altered cognitive ability. Decreases in learning potential are reported during adolescence, where puberty onset may represent the end of a critical period for optimal learning for some types of basic tasks (Johnson and Newport, 1989; Subrahmanyam and Greenfield, 1994), an effect which can be more pronounced in females (Hassler, 1991). Synaptic plasticity assessed using long-term potentiation (LTP), an *in vitro* model of learning, is also impaired at puberty in the female mouse (Shen et al., 2010). These changes in cognition cannot readily be explained by the fluctuations in pubertal hormones at this time (Smith, 2013). However, cognition is impacted by the degree of inhibitory tone in areas that underlie learning, such as the CA1 hippocampus. Synaptic inhibition in the hippocampus is provided by a wide array of GABAergic interneurons which serve to sculpt neuronal circuits. Of these, the parvalbumin-containing (PV+) interneurons, which generate 40 Hz gamma oscillations in addition to providing potent inhibition to the circuit, play an important role in cognition (Buzsáki and Wang, 2012). This article will test whether PV+ interneuron-generated synaptic current increases at puberty and if this increase in inhibitory current contributes to the impairment in synaptic plasticity observed at this time. Our previous findings showed that the increase in tonic inhibitory current generated by extrasynaptic α4βδ GABARs at puberty (Shen et al., 2007) plays a role in impairing synaptic plasticity and spatial learning.

PV expression increases during development in the CA1 hippocampus (Wu et al., 2014) and medial prefrontal cortex (mPFC; Caballero et al., 2014a). However, PV+ interneuron number is not increased (Baker et al., 2017), implicating growth and proliferation of PV+ neurites in mPFC during adolescence. It is not known if the dendrite branching of PV+ interneurons in the CA1 hippocampus is altered by puberty onset.

The activity of PV+ interneurons is affected by ovarian hormones during pregnancy, raising the possibility that ovarian hormonal changes at puberty onset in the female mouse may have a similar effect. PV+ interneurons in both CA1 and CA3 hippocampus express the δ GABA_A_ receptor (GABAR) subunit (Ferando and Mody, 2013), which co-expresses with α1 rather than α4 (Glykys et al., 2007), a more common expression pattern (Wei et al., 2003). δ expression on the PV+ interneurons is decreased during pregnancy (Ferando and Mody, 2013). This study will examine if similar changes in δ expression on PV+ interneurons occur at puberty as one factor which may alter the output from these inhibitory interneurons.

Therefore, this study examined changes in the phasic current generated by PV+ interneurons in the CA1 hippocampus before and after the onset of puberty in female mice. Two potential mechanisms for changes in PV+ interneuron-generated current were also examined during adolescence: dendrite branching of PV+ interneurons and δ GABAR expression on these neurons. sIPSC characteristics were examined in mice with reduced δ expression to determine the role of δ-GABARs in regulating PV+ interneuron activity. The impact of alterations in PV+ interneuron-generated inhibition on the induction of LTP was also tested. Also, we examined the effect of the positive GABA modulator/stress steroid THP (3α-OH, 5α-pregnan-20-one; Purdy et al., 1991) on the phasic current. The findings from this study will determine whether the phasic current impairs synaptic plasticity and the response to a stress hormone during adolescence like the tonic current (Shen et al., 2007).

## Materials and Methods

### Animal Subjects

Female wild-type C57BL/6 mice (Jackson Labs, Bar Harbor, ME, USA), GAD67-GFP mice, and GABAR δ+/− mice were used. Hemizygous GAD67-GFP mice (Jackson Labs, Bar Harbor, ME, USA, line G42, stock#007677, CB6-Tg (Gad1-EGFP)G42Zjh/J), which selectively express an enhanced green fluorescent protein (EGFP) in the calcium-binding protein parvalbumin (PV)-expressing subclass of basket interneurons, were bred on-site to yield mice that had high expression of GFP, as revealed by genotyping results (Transnetyx, Cordova, TN, USA). Only mice with high expression of GFP were used to trace the neuronal processes; lower expressing mice were excluded from the study. δ+/− mice were used rather than δ−/− mice because of the reduced breeding capacity of the homozygous knock-out and were bred on site. Mice were used at pre-pubertal (PND 25–34) and pubertal (~beginning at puberty onset, ~PND 35, confirmed by vaginal opening, -PND 44) ages. Mice were housed in a reverse light:dark cycle (12:12) and euthanized in the light part of the cycle 1 h before dark onset. This study was carried out following the principles of the National Institute of Health Office of Laboratory Animal Welfare (OLAW) and recommendations of the SUNY Downstate Institutional Animal Care and Use Committee (IACUC). The protocol was approved by the SUNY Downstate IACUC before the study was initiated.

### Fluorescence Microscopy and Immunohistochemistry

#### Preparation of Brains

GAD67-GFP mice were perfused under urethane (0.1 ml 40%) anesthesia first with 100–300 ml of heparinized saline over a 1-min period followed by 4% paraformaldehyde in 0.1 M phosphate buffer (PB), set at pH 7.4, over a 1–2-min period. Brains were post-fixed for 24 h before sectioning at 35 μm (immunohistochemistry) or 100 μm (Scholl analysis) on a vibratome (Leica VT1200S). The sections were stored at 4°C in saline (0.9% NaCl), buffered by 0.01 M phosphate salts (PBS, pH 7.4) and with 0.05% sodium azide.

#### Morphological Analysis

PV+ interneurons were visually identified in CA1 using confocal microscopy, based on their green fluorescence, which in these transgenic mice is selective for PV+ interneurons. Only mice with high expression of GFP, as revealed by genotyping results, were used for the study. Other reports show that an increased GFP signal in these mice allows for accurate analysis of dendritic branching (Curto et al., 2019). Imaged sections were 100 μm thick, and cells were only analyzed if the soma was located centrally, at roughly 50 μm (±5 μm). On average, 3–5 neurons from both hemispheres of the dorsal hippocampus were included per animal, with 4–6 animals in each group (25 neurons per group). Image stacks (50; 1–2 μm thick) of the neurons that met the criteria were recorded with an Olympus FluoView TM FV1000 confocal inverted microscope with objective UPLSAPO 40× NA:1:35 (Olympus, Tokyo, Japan). Neuron tracing was performed using the semi-automated, “Simple neurite tracer” plugin (Longair et al., 2011), for the open-source program, Fiji (Schindelin et al., 2012) an automated program for direct analysis of fluorescent images. Sholl analysis was conducted using the “3DSholl analysis” plugin^1^, based on the number of intersections between dendrites and the surface of spheres with a radius increment of 10 μm (Ferreira et al., 2014). Dendritic branching was evaluated for primary, secondary, tertiary, and higher (quaternary and quinary) levels.

#### Immunohistochemistry (Afroz et al., 2016)

The sections were blocked in 0.01 M PBS supplemented with 10% donkey serum, 0.4% Triton and 0.05% sodium azide for 2 h at room temperature. Then, sections were incubated with rabbit anti-GABRD (ab110014, Abcam, 1:200) for 24 h at 4°C. After washing, sections were incubated with secondary antibody (Alexa fluor 568 donkey anti-rabbit) overnight at 4°C. For the negative control, the primary antibody was omitted. All slices were mounted on slides with ProLong Gold Antifade Reagent. Images were taken with an Olympus FluoView TM FV1000 confocal inverted microscope with objective UPLSAPO 60× NA:1:35 (Olympus, Tokyo, Japan) after adjusting the laser intensity to minimize background. Images were analyzed for luminosity at 568 λ (reflecting GABAR δ expression) using the region of interest (ROI) program of Fiji (Image J) software (NIH). PV+ interneurons were identified by their GFP signal (488 λ). All images presented are optimized to an equivalent degree.

### Electrophysiology

#### Hippocampal Slice Preparation

Following decapitation of wild-type C57BL/6 mice, the brains were removed and cooled using an ice-cold solution of artificial cerebrospinal fluid (aCSF) containing (in mM): NaCl 124, KCl 5, CaCl_2_ 2, KH_2_PO_4_ 1.25, MgSO_4_ 2, NaHCO_3_ 26, and glucose 10, saturated with 95% O_2_, 5% CO_2_ and buffered to a pH of 7.4. Brains were then sectioned at 400 μm on a Leica microtome (VT1000S) and slices incubated (1 h) in oxygenated aCSF.

#### Whole-Cell Patch-Clamp Electrophysiology (Shen et al., 2007)

CA1 hippocampal pyramidal cells were visualized using a Leica differential interference contrast (DIC)-infrared upright microscope. Whole-cell patch-clamp recordings were carried out in voltage-clamp mode at a holding potential of −50 or −60 mV at room temperature (Axopatch 200B amplifier, Axon Instruments, 20 kHz sampling frequency, 2 kHz 4-pole Bessel filter) and pClamp 9.2 software. Patch pipets were fabricated to yield open tip resistances of 2–4 MΩ. Electrode capacitance and series resistance were monitored and compensated; access resistance was monitored throughout the experiment, and cells discarded if the access resistance >10 MΩ. Five millimolar QX-314 was added to the pipet solution to block voltage-gated Na+ channels and GABA_B_ receptor-activated K+ channels (Nathan et al., 1990; Otis et al., 1993). The bath contained 2 mM kynurenic acid and 1 μM strychnine to pharmacologically isolate the GABAergic current.

##### sIPSC Recordings, Pre, and Post-puberty

The pipet solution (in mM) was: CsCl 140, HEPES 5, EGTA 5, Mg-ATP 2, CaCl_2_-H_2_O 0.5, QX-314 5 (Calbiochem), Li-GTP 0.5, pH 7.2, 290 mOsm (holding potential, −60 mV).

##### sIPSC Recordings, Polarity Dependent Effects of THP

The pipet solution (in mM) for E_Cl_ = −70 (or E_Cl_ = −30) was: K-gluconate 141.5 (0), KCl 0 (52.3), CsCl 8.5 (145), HEPES 5, EGTA 5, CaCl_2_-H_2_O 0.5, QX-314 5 (Calbiochem), Mg-ATP 2, Li-GTP 0.5, pH 7.2, 290 mOsm (holding potential, −50 mV).

##### sIPSC Analysis

sIPSCs were detected using a threshold delimited event detection sub-routine in pClamp11.0.3. Only data with a stable baseline were included in the analysis. Synaptic events (200–300) were averaged for each treatment condition and the current amplitude, half-width, and sIPSC frequency assessed. In some cases, histograms were constructed for sIPSC peak amplitude across pubertal groups.

#### Long-Term Potentiation (LTP; Shen et al., 2010)

The CA1 hippocampal pyramidal cell layer was visualized using an upright microscope. Field excitatory postsynaptic potentials (fEPSPs) were recorded extracellularly from the stratum radiatum of CA1 hippocampus using an aCSF-filled glass micropipette (1–5 mΩ) in response to stimulation of the Schaffer collateral-commissural pathway using epoxylite insulated platinum-iridium parallel (150 μm distance) bipolar electrodes (57 mm long, 50–100 KΩ impedance, FHC, Bowdoin, ME, USA). The intensity of the stimulation was adjusted to produce 50% of the maximal response. LTP was induced using theta-burst stimulation (TBS, 8–10 trains of four pulses at 100 Hz, delivered at 200 ms intervals, repeated 3× at 30 s intervals) which is a physiological stimulation pattern. fEPSP responses were recorded at 30 s intervals with an Axoprobe-1A amplifier and pClamp 10.1 for 20 min. before and 120 min. after TBS (producing 1–4 mV EPSPs). In some cases, the μ-opioid agonist DAMGO (1 μM) was bath applied before LTP induction to block PV+ interneurons (Gulyás et al., 2010).

### Drugs

#### DAMGO and WINN

In some cases, the μ-opioid agonist DAMGO [1 μM, (D-Ala^2^, N-MePhe^4^, Gly-ol)-enkephalin] or the CB1 cannabinoid receptor agonist WINN (1 μM, WIN 55, 212-2, *R*)-(+)-[2,3-Dihydro-5-methyl-3-(4-morpholinylmethyl) pyrrolo[1,2,3-*de*]-1,4-benzoxazin-6-yl]-1-naphthalenylmethanonemesylate) were bath applied to silence PV+ or CCK+ interneurons, respectively (Svoboda et al., 1999; Hájos et al., 2000; Gulyás et al., 2010). Both drugs were from Tocris Bioscience, Minneapolis, MN, USA.

#### THP

In some cases, the neurosteroid THP (3α-OH, 5α-pregnan-20-one, Steraloids, Newport, RI, USA) was bath applied during sIPSC recording. THP was used at a physiological concentration (30 nM) as hippocampal levels vary between 15 nM and 50 nM (Smith et al., 2006).

### Statistics

All data are represented by the mean ± standard error of the mean (SEM). Initially, the Kolmogorov–Smirnov test was used to confirm that all data followed a normal distribution, and then the specific comparisons (see below) were undertaken. For all statistical tests, the level of significance was determined to be *P* < 0.05.

#### Synaptic Current

Planned comparisons for changes in GABA-gated current before and after drug treatment for the same cell were analyzed using a paired *t*-test. sIPSC comparisons from pre-pubertal and pubertal groups were tested using the student’s *t*-test.

#### LTP

A statistically significant potentiation at 120 min. in the LTP study was determined by averaging the final 20 values for EPSP slope after TBS compared with the average pre-TBS values using the paired *t*-test. Comparisons of these final 20 slope values between experimental groups were accomplished with a student’s *t*-test.

#### Dendritic Branching

For each assessment, the means of 3–6 neurons from each mouse were used. For the Scholl analysis, branching assessed at 10 μm intervals was compared in pre-pubertal vs. pubertal groups using the student’s *t*-test. Also, branching of primary, secondary, tertiary, and quaternary dendrites were assessed separately comparing pre-pubertal vs. pubertal groups using the student’s *t*-test.

#### Immunohistochemistry

Mean values of δ luminosity/mouse were averaged from six ROI’s per mouse, and then mean group values averaged for pre-pubertal and pubertal mice. A one-tailed *t*-test was used to test the hypothesis that δ expression is decreased at puberty.

## Results

### Inhibitory Synaptic Current Is Increased at Puberty in CA1 Hippocampus

**Figure 1:**
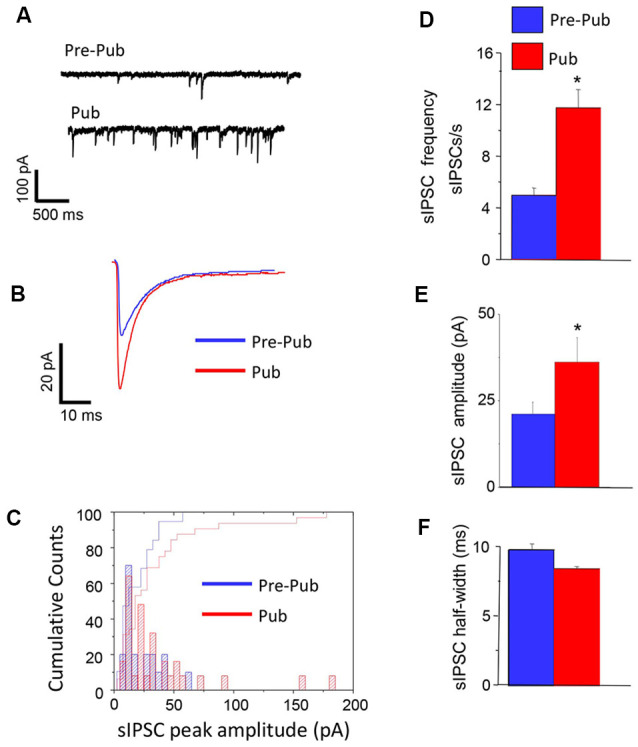
Inhibitory synaptic current is increased at puberty in the CA1 hippocampus. Spontaneous inhibitory postsynaptic currents (sIPSCs), recorded with whole-cell patch-clamp techniques from CA1 hippocampal pyramidal cells at a −60 mV holding potential from pre-pubertal (Pre-Pub) and pubertal (Pub) female mice. **(A)** Representative traces. **(B)** Averaged currents. **(C)** Histogram, peak current amplitude distribution. **(D–F)** Averaged values, sIPSC frequency, amplitude, and half-width. Pre-pub vs. pub: frequency, *t*_(20)_ = 3.22, *P = 0.0021; amplitude, *t*_(43)_ = 1.9, *P < 0.03, *n* = 10–12 cells/group.

We recorded the pharmacologically isolated GABAergic synaptic current using whole-cell patch-clamp techniques in the hippocampal slice before and after the onset of puberty. The frequency of spontaneous inhibitory postsynaptic currents (sIPSCs) was increased by more than 2-fold at puberty compared to pre-pubertal values (*P* = 0.0021, Figure 1). In addition, the mean peak amplitude of these currents was also increased by ~60% at puberty (*P* < 0.03), unaccompanied by significant changes in the sIPSC half-width. These data suggest that sIPSCs, which reflect compound action potential-dependent and action potential-independent synaptic currents, are increased at puberty in the CA1 hippocampus of the female mouse.

### Pharmacological Identification of Interneuron Sub-type

**Figure 2:**
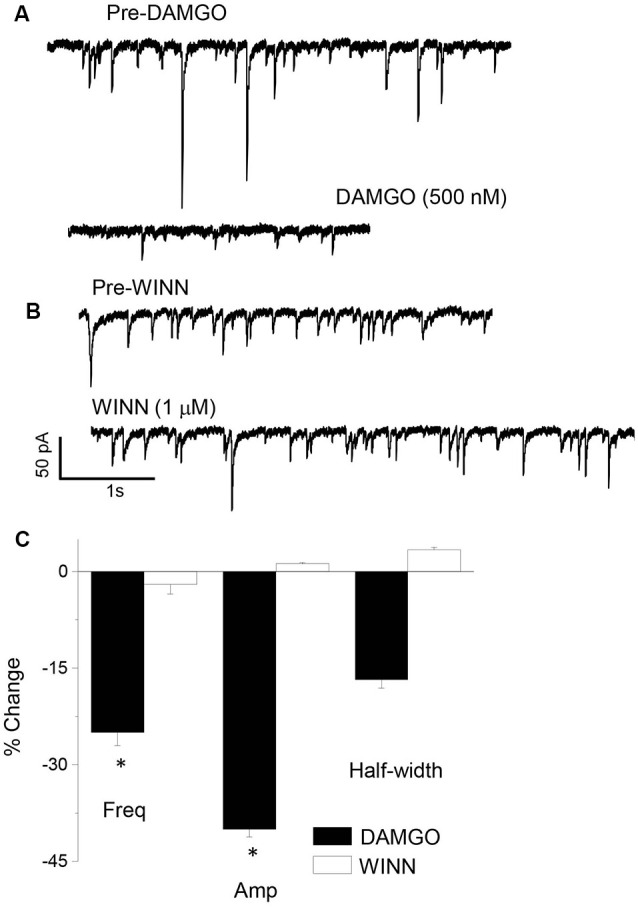
sIPSCs generated in the pubertal hippocampus are reduced by DAMGO. Representative sIPSCs recorded before (top traces) and after (bottom traces) bath application of 1 μM DAMGO **(A)** or 1 μM WINN 55, 212-2 (WINN, **B**) to silence PV+ or CCK-containing interneurons, respectively. **(C)** Averaged data showing the percentage change in sIPSC frequency (Freq), amplitude (Amp), and half-width induced by the two drugs. 1 μM DAMGO, frequency, *t*_(8)_ = 1.97, *P = 0.042; amplitude, *t*_(8)_ = 10.7, *P < 0.00001, *n* = 5 cells/group.

There is a diverse group of interneurons localized to the CA1 hippocampus, which is characterized by unique intracellular markers such as parvalbumin (PV) or cholecystokinin (CCK). We used a pharmacological approach to distinguish between these interneuron populations for their role in generating sIPSCs at puberty. sIPSCs which are generated from PV+ interneurons can be blocked with 1 μM DAMGO, while those generated by CCK-containing interneurons are significantly reduced with 1 μM WINN 55, 212-2 (WINN; Hájos et al., 2000; Glickfeld et al., 2008; Gulyás et al., 2010; Katona et al., 1999). We employed this strategy to determine the origin of sIPSCs which are increased in frequency in the pubertal hippocampus. To this end, the drugs were bath applied in different recordings of sIPSCs and results compared with the pre-drug control period. DAMGO, but not WINN, reduced sIPSC frequency by 25% (*P* = 0.042, Figure 2) while reducing sIPSC amplitude by 40% (*P* < 0.00001). These results suggest that PV+ interneurons contribute significantly more GABAergic input during the pubertal period than the corresponding contribution from CCK-containing interneurons.

### Expression of the GABAR δ Subunit Is Decreased at Puberty on PV+ Interneurons in CA1 Hippocampus

**Figure 3:**
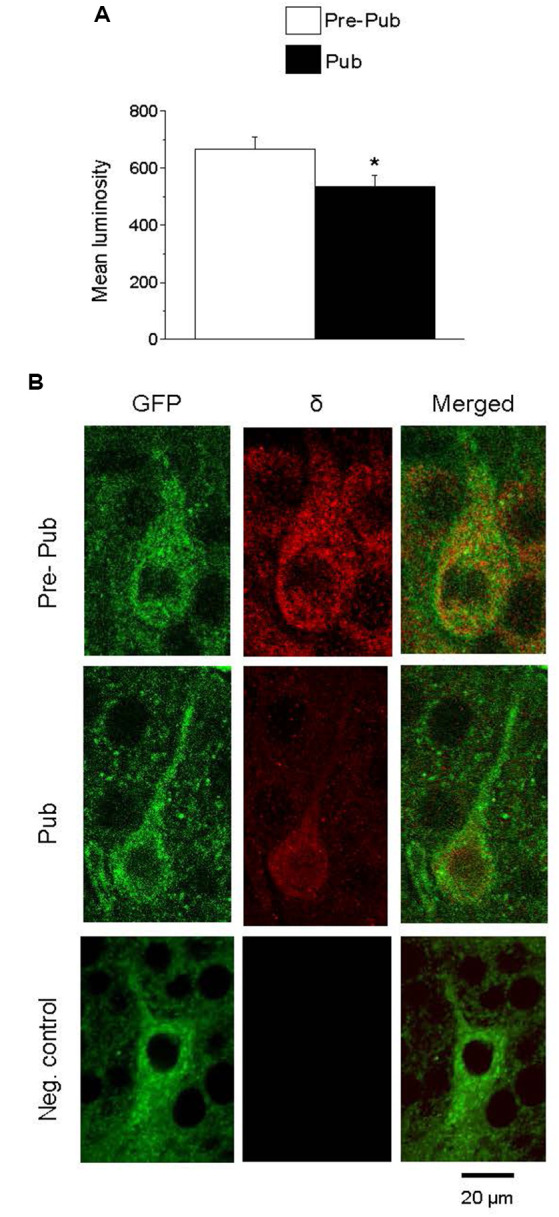
The expression of the GABA_A_ receptor (GABAR) δ subunit on PV+ interneurons is decreased at puberty in the CA1 hippocampus. **(A)** Averaged data depicting mean values of luminosity for GABAR δ immunofluorescence. **(B)** Representative images of the CA1 hippocampal pyramidal cell layer depicting PV+ interneurons: GFP (488 λ), δ staining (568 λ) and merged, imaged at 40×, from a pre-pubertal (Pre-Pub) and pubertal (Pub) mouse as well as a negative control (neg. control) with the primary antibody omitted. *t_(8)_ = 2.28, *P* = 0.026. *n* = 5 mice/group averaged from six neurons/mouse.

Recent studies have reported the expression of α1βδ GABARs on PV+ interneurons in both CA1 and CA3 hippocampus (Ferando and Mody, 2013). Expression of these receptors is decreased by ovarian hormones which are increased during pregnancy (Ferando and Mody, 2013). For the present study, we tested whether the ovarian hormonal changes at puberty would also decrease δ expression on PV+ interneurons in the CA1 hippocampus of the female mouse. To this end, immunohistochemical techniques were used to assess the staining of the GABAR δ subunit on PV+ interneurons from GAD67-GFP mice. PV+ interneurons were identified by their selective GFP stain. δ expression was significantly decreased at puberty compared to pre-puberty (Figure 3, 667.1 ± 43.7, pre-pub vs. 536.15 ± 37, pub, *P* = 0.026) suggesting that tonic inhibition of these interneurons is reduced at puberty. A negative control is presented showing no staining with the secondary antibody when the primary antibody is omitted (Figure 3B).

### sIPSC Frequency and Amplitude Are Increased Before Puberty in Mice With Reduced δ Expression

**Figure 4:**
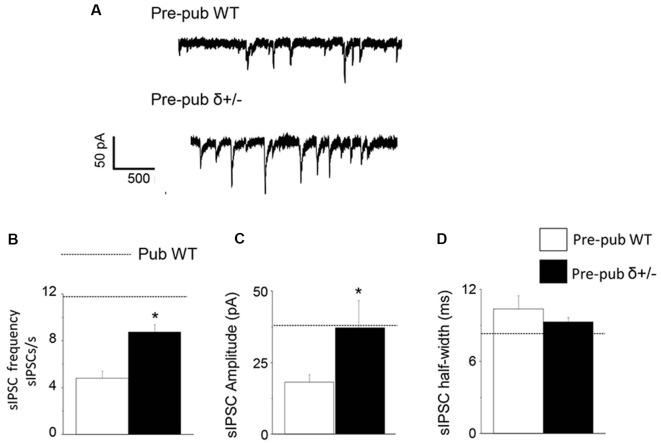
δ knock-out increases sIPSC frequency and amplitude in the pre-pubertal CA1 hippocampus.** (A)** Representative traces, sIPSCs recorded from pre-pubertal wild-type (upper trace) or δ+/− (lower trace) CA1 hippocampal pyramidal cells. **(B–D)** Averaged data depicting mean values for sIPSC frequency, amplitude, and half-width. Dashed lines indicate average values from pubertal mice. Pre-Pub WT vs. Pre-Pub δ+/−: frequency, *t*_(20)_ = 5.38, *P = 0.000028; amplitude, *t*_(75)_ = 1.77, *P < 0.04, *n* = 11 cells/group.

Because the high levels of GABAR δ subunit expression on PV+ interneurons decrease at puberty in association with increases in sIPSC frequency and amplitude, we tested whether reducing δ expression would increase sIPSC frequency and amplitude in pre-pubertal CA1 hippocampus. To this end, sIPSCs were recorded from CA1 hippocampal pyramidal cells from wild-type and δ+/− mice. In the pre-pubertal mouse hippocampus with reduced δ expression, sIPSC frequency and amplitude were increased almost 2-fold compared to wild-type (Figure 4, *P* < 0.00028, and *P* < 0.04, respectively). In contrast, sIPSC half-width was not significantly different. Both amplitude and half-width were not significantly different from pubertal values, although frequency was significantly lower (*t*_(19)_ = 2.47, *P* = 0.023). These data suggest that reduced δ expression on PV+ interneurons can increase sIPSC frequency and amplitude on CA1 hippocampal pyramidal cells recorded before puberty.

### Dendritic Branching of PV+ Interneurons Is Increased at Puberty

**Figure 5:**
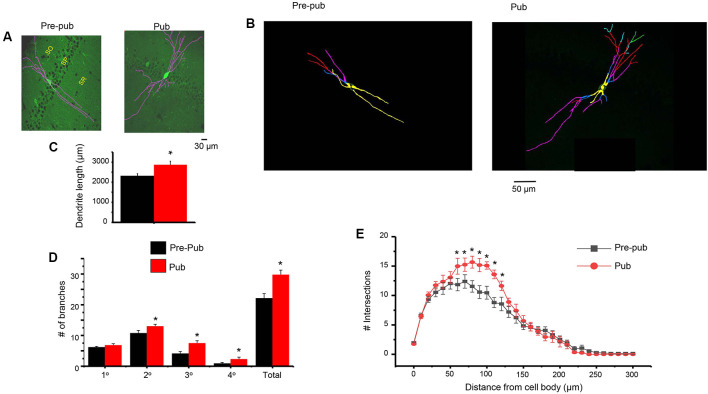
Dendritic branching of PV+ interneurons in CA1 hippocampus is increased at puberty. **(A)** Representative images (Image J), PV+ interneurons in the CA1 hippocampal pyramidal cell layer, pre-pub and pub. SO, stratum oriens; SP, stratum pyramidale; SR, stratum radiatum. **(B)** Representative images (Neurolucida tracing), indicating levels of branching for the interneurons in panel **(A)**. Yellow, 1°; blue, 2°; pink, 3°; red, 4°; green, 5°. **(C)** Dendrite length. Pre-pub vs. pub: *t_(12)_ = 2.6, *P* = 0.012. *n* = 6–7 mice (3–6 neurons each). **(D)** Number of branches for different classes of dendrites, pre-pub vs. pub: primary, *t*_(12)_ = 1.48, *P* = 0.083 ; secondary, *t_(12)_ = 2.0, *P* = 0.034; *tertiary, *t*_(12)_ = 3.45, *P* = 0.0024; *quaternary (includes quinary), *t*_(12)_ = 2.23, *P* = 0.00227; *total, *t*_(12)_ = 3.64, *P* = 0.0017. **(E)** Sholl analysis, the number of dendritic crossings assessed across the apical dendrite. Pre-pub vs. pub: *80 μm, *t*_(11)_ = 2.91, *P* = 0.0142; *90 μm, *t*_(11)_ = 2.92, *P* = 0.014; *100 μm, *t*_(11)_ = 3.47, *P* = 0.005; *110 μm, *t*_(11)_ = 4.16, *P* = 0.00159.

One potential mechanism for the increase in sIPSC frequency attributable to PV+ interneurons at puberty would be an increase in inputs from these interneurons. Therefore, we examined whether the dendritic branching of PV+ interneurons is altered during puberty. To this end, confocal microscopy was used to capture images of PV+ interneurons taken from GAD67-GFP mice at the prepubertal and pubertal stages. Scholl analysis was used to quantify branching across the length of the dendrite. Dendritic branching was significantly increased at puberty compared to pre-puberty when assessed 80 (*P* = 0.0142), 90 (*P* = 0.014), 100 (*P* = 0.005), and 110 (*P* = 0.00159) μm from the cell body (Figure 5). When different classes of dendrites were evaluated, pubertal branching was significantly greater (Figure 5) for secondary (*P* = 0.034), tertiary (*P* = 0.0024), and quaternary/quinary dendrites (*P* = 0.00227), but not for primary dendrites. Total branches (*P* = 0.0017) and dendrite length (*P* = 0.012) were also greater at puberty compared to pre-puberty.

### The Neurosteroid THP Does Not Alter sIPSCs at Puberty in CA1 Hippocampus

**Figure 6:**
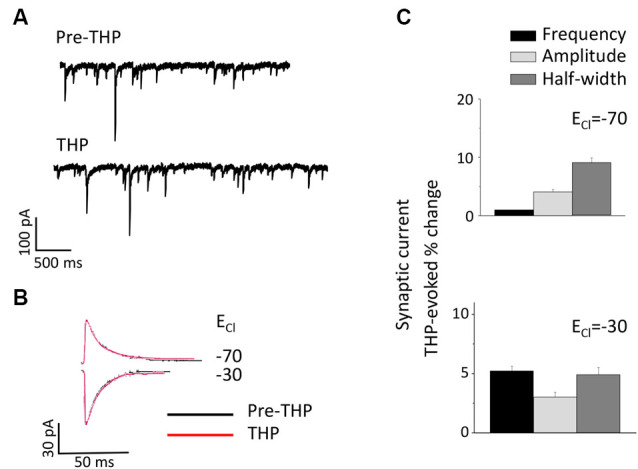
The neurosteroid THP does not affect pubertal sIPSCs recorded in the CA1 hippocampus. **(A)** Representative traces before and after 30 nM THP. **(B)** Averaged currents before and after 30 nM THP, recorded at a −50 mV holding potential. The internal Cl^−^ concentration was varied to achieve an E_Cl_^-^ of −70 mV (top) or −30 mV (bottom). **(C)** Averaged values for the effect of THP on sIPSC amplitude, half-width, and frequency for outward (upper panel) and inward (lower panel) current. *n* = 8–10 cells/group.

THP is a metabolite of the ovarian hormone progesterone and is also formed and released in the brain after prolonged stress (Purdy et al., 1991) when it typically enhances inhibition (Bitran et al., 1999; Stell et al., 2003). Its effects on the tonic current generated by extrasynaptic α4βδ GABARs are polarity-dependent, where it decreases outward current but increases inward current (Shen et al., 2007). Thus, we tested whether this steroid has effects on the phasic GABAergic current at puberty and further whether polarity-dependent effects could be observed. To this end, the direction of the phasic current was reversed by altering [Cl^−^]_I_ so that THP effects could be tested on both outward (E_Cl_ = −70 mV) and inward (E_Cl_ = −30 mV) current. Unlike its effects on the tonic current, THP did not significantly alter any sIPSC parameter for current in either direction (Figure 6).

### Silencing PV+ Interneurons Restores Induction of LTP Using Theta-Burst Stimulation at Puberty

**Figure 7:**
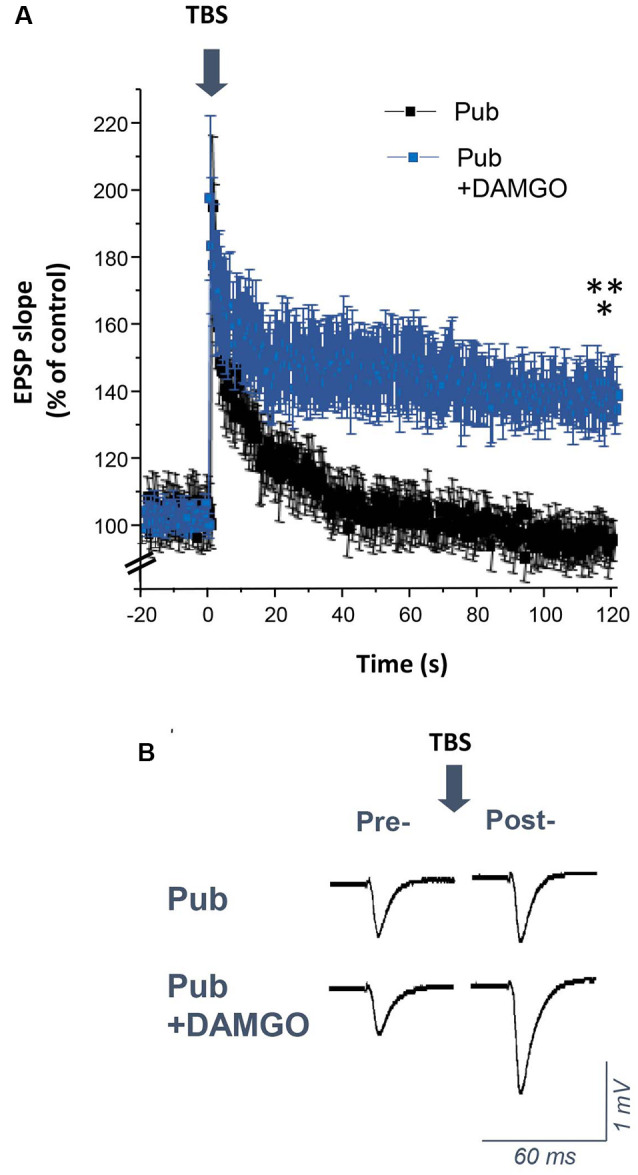
DAMGO restores the induction of long-term potentiation (LTP) using theta-burst stimulation (TBS) in the CA1 hippocampus at puberty. **(A)** Averaged values (mean ± SEM) for the % change in EPSP slope as a percentage of control, recorded for 20 min before and 2 h after theta-burst stimulation (TBS, arrow), recorded with or without 1 μM DAMGO. **(B)** Representative fEPSPs before and after TBS, recorded with or without 1 μM DAMGO. Pub+DAMGO, *t*_(7)_ = 5.01, *p = 0.0016 vs. pre-TBS baseline; *t*_(15)_ = 3.2, **p = 0.00597 vs. Pub, *n* = 8 slices/group. Arrow, TBS.

Induction of LTP by TBS is impaired at puberty despite robust LTP induction observed pre-pubertally (Shen et al., 2010). In this study, we tested whether increased phasic inhibition from the PV+ interneurons contributes to the impairment in TBS-induction of LTP at puberty. To this end, TBS of the Schaffer collaterals was used to induce LTP in the CA1 hippocampal slice (Figure 7). As noted previously, this method was unsuccessful in inducing LTP in pubertal slices (Shen et al., 2010). However, bath application of 1 μM DAMGO to silence the PV+ interneurons restored LTP induction (Figure 7, *P* = 0.0016), resulting in an EPSP slope 150% of control, significantly greater than pubertal values (*P* = 0.00597). These findings suggest that phasic inhibition from PV+ interneurons, which increases at puberty, plays a role in the deficits in synaptic plasticity observed at this time.

## Discussion

The results from the present study demonstrate a significant increase in inhibitory current recorded from CA1 pyramidal neurons at the onset of puberty in female mice. PV+ interneurons contributed significantly to this increase in the phasic current. These interneurons, identified by their selective GFP expression in transgenic mice, displayed greater dendritic branching at puberty and decreased expression of the GABAR δ subunit, reflecting disinhibition of these neurons. The pubertal increase in phasic inhibition *via* PV+ interneurons impaired synaptic plasticity, assessed by the induction of LTP.

DAMGO blocks GABA release from PV+ fast-spiking basket cells (Gulyás et al., 2010) *via* the μ-opioid receptors which express on these interneurons (Svoboda et al., 1999). Although the original article showing this effect (Gulyás et al., 2010) used carbachol-activated PV+ interneurons, the reduction in tonic inhibition of the PV+ interneurons due to the decrease in α1βδ GABARs would similarly activate these interneurons. However, the effect of DAMGO is not entirely specific, as this drug has a small inhibitory effect on regular spiking basket cells (Glickfeld et al., 2008) and can also inhibit ivy and neurogliaform cells (Krook-Magnuson et al., 2011; Harris et al., 2018). All of these interneurons are slow firing and exert relatively slower effects than the PV+ interneurons (Krook-Magnuson et al., 2011; Overstreet-Wadiche and McBain, 2015). Therefore, the majority of the higher frequency IPSCs which are blocked by DAMGO would be expected to originate in the PV+ interneurons. DAMGO reduced by about half the increased IPSC frequency seen at puberty but reduced more completely the increased IPSC amplitude seen at puberty. These findings suggest that other interneurons may also contribute to the pubertal increase in IPSC frequency, while the larger amplitude IPSCs are likely generated by the PV+ interneurons.

The increase in dendritic branching of PV+ interneurons at puberty could result in increased inhibitory synaptic input to the pyramidal cells and may underlie the observed increase in phasic current at puberty, although other possibilities such as presynaptic effects and altered PV+ activity, as discussed below, may play a role. Increases in PV expression have been reported in the hippocampus across development (Wu et al., 2014) unaccompanied by increases in interneuron numbers (Honeycutt et al., 2016), although puberty has not been investigated until the present study. Our results showing an increase in dendritic branching at puberty may underlie these findings. Similar findings in mPFC suggest that increased PV expression (Caballero et al., 2014b; Caballero and Tseng, 2016) unaccompanied by changes in interneuron density (Baker et al., 2017) are correlated with increases in synaptic current (Caballero et al., 2014b) and may also reflect an increase in dendritic branching.

Recent studies suggest that GABAergic input to hippocampal interneurons is depolarizing, but becomes a shunting inhibition at high conductances (Song et al., 2011). α1βδ GABARs express close to the soma of PV+ interneurons (Glykys et al., 2007) where GABAR inhibition is shunting (Elgueta and Bartos, 2019) unlike the hyperpolarizing inhibition on the distal dendrites which may have more of a role in reducing sub-threshold events. The high circulating levels of THP before puberty (Mannan and O’Shaughnessy, 1988) would amplify inhibition generated by these α1βδ GABARs, where up to ~20-fold increases have been reported (Zheleznova et al., 2008), and would ensure that α1βδ GABARs generate a tonic inhibition to be effective at silencing PV+ interneurons. At puberty, α1βδ GABAR expression on these interneurons decreases as do hippocampal levels of THP (~60%; Shen et al., 2007). We can extrapolate from our previous findings (Shen et al., 2007) to estimate that the 20% decrease in δ expression at puberty in the present study would decrease charge transfer by 2.3% given the low open probability of α1βδ GABARs in the absence of neurosteroid (Bianchi et al., 2002; Zheleznova et al., 2008). However, because neurosteroid levels decrease at puberty from high pre-pubertal levels which would amplify tonic inhibition before puberty, the decrease in charge transfer could be as much as 78%. These events would disinhibit interneuron firing, resulting in increased phasic inhibition of the pyramidal cells.

Our current findings support a role for α1βδ GABARs on PV+ interneurons in regulating interneuron activity because sIPSC frequency and amplitude were increased to pubertal levels in pre-pubertal mice with reduced δ expression. This finding confirms the functional significance of the inhibition generated by the α1βδ GABARs which express on the PV+ interneurons. Although δ GABARs can contribute to the phasic current of the principal cells (Sun et al., 2018), this may be a minimal effect compared to their effect on interneuron activity. Taken together, these data provide additional evidence that decreases in δ expression at puberty disinhibit PV+ interneurons and likely contribute to the pubertal increase in sIPSC frequency and amplitude.

The decrease in δ expression on, and thus decrease in tonic inhibition of, PV+ interneurons at puberty may be a mechanism for the profuse dendritic branching observed at this time, as dendritic growth is dependent upon NMDA receptor-induced activity (Sepulveda et al., 2010). Our previous findings show that in the CA3 hippocampus, knock-out of α4βδ GABARs increases the dendritic branching of CA3 pyramidal cells (Parato et al., 2019), suggesting a role for these receptors in limiting dendritic arborization. Other factors that may contribute include the increase in 17β-estradiol (E2) around the time of puberty (Ahima et al., 1997), as E2 has been shown to increase dendritic branching of hippocampal neurons (Audesirk et al., 2003). There are multiple potential mechanisms for this effect, including E2’s effect to increase levels of brain-derived neurotrophic factor (BDNF; Scharfman et al., 2007) which on its own can directly increase dendritic growth and arborization in many CNS areas (Horch and Katz, 2002), including the hippocampus (Cheung et al., 2007), an effect mediated by cyclin-dependent kinase 5 (Cdk5). The effects of BDNF are rapid, with significant branching seen after 48 h (Sanchez et al., 2006). E2 has also been shown to increase dendritic branching in the hippocampus *via* nitric oxide (Audesirk et al., 2003).

Increased branching of PV+ interneurons in the CA1 hippocampus at puberty would enhance synaptic contacts with the principal cells and effectively increase inhibitory tone. In the present study, we show that this increase in inhibition at puberty is one factor leading to impairment of synaptic plasticity at puberty, when induction of LTP using TBS is mostly unsuccessful (Shen et al., 2010). Silencing PV+ interneurons with DAMGO restored TBS-induced LTP, suggesting that the increase in phasic inhibition mediated by these interneurons plays a role in impairing synaptic plasticity at puberty. Recent studies (Camiré et al., 2018; Topolnik and Camiré, 2019) suggest that TBS can also impact the plasticity of PV+ interneurons. In young (PND 13–21) mice, sub-threshold TBS induced LTP and supra-threshold TBS induced LTD in PV+ interneurons, which reverted to LTP after blockade of supralinear Ca++ signals. Because these mice have very low expression of δ-GABARs (Laurie et al., 1992) it is possible that at puberty when δ expression is increased, TBS may increase synaptic strength of PV+ interneurons as one additional factor to impair synaptic plasticity.

In addition, based on previous reports (Ferando and Mody, 2015) showing that reduced α1βδ expression increases the frequency of gamma oscillations, the pubertal decrease in α1βδ expression would be expected to result in a similar change in gamma frequency, which may also be detrimental to cognition, *in vivo* (Buzsáki and Watson, 2012).

Our previous study showed that the tonic inhibition generated by α4βδ GABARs, which emerge on the dendritic spines of CA1 pyramidal cells at puberty (Shen et al., 2010) from nearly undetectable levels before puberty (Shen et al., 2007), also play a critical role in the impairment of TBS-induced LTP at puberty. Knock-out of these receptors restores LTP induction, suggesting that increases in both tonic and phasic inhibition at puberty contribute to the deficits in synaptic plasticity at this time. The increase in interneuron activity at puberty could also enhance tonic inhibition via GABA spillover. Other studies have shown impairments in spike-timing-dependent plasticity at puberty that was due to increased GABAergic inhibition (Meredith et al., 2003). Knock-out of α4βδ GABARs also restores optimal levels of spatial learning at puberty (Shen et al., 2010, 2017), when this type of learning is impaired compared to pre-puberty. Independent of puberty, the inhibitory current generated by α5-GABARs hinders synaptic plasticity in the CA1 hippocampus, by raising the threshold for induction of LTP and impairing certain types of hippocampal-dependent memory *in vivo* (Martin et al., 2010). In addition to their extrasynaptic localization, recent studies have shown that α5-GABARs also express sub-synaptically where they are the target of somatostatin-containing interneurons on pyramidal cells (Schulz et al., 2018) as well as on somatostatin-containing interneurons, where they disinhibit the circuit (Magnin et al., 2019). In the former case, these outwardly rectifying receptors efficiently reduce NMDAR-generated potentials. Although, they may play a role in synaptic plasticity under some conditions, blockade of α5-GABARs at puberty does not restore LTP induction using TBS (Shen et al., 2017).

In human studies, the onset of puberty is known as a critical time-point when certain types of learning are sub-optimal compared to the rapid learning observed before puberty (Gur et al., 2012). Post-pubertal impairments are reported for learning a second language, music training and certain visuospatial tasks (Pepin and Dorval, 1986; Johnson and Newport, 1989; Subrahmanyam and Greenfield, 1994; Shavalier, 2004; Bailey and Penhune, 2012), which are more pronounced in individuals with learning disabilities (Wright and Zecker, 2004) and also exhibit gender differences (Gur et al., 2012). Increased GABAergic tone, mediated by both phasic and tonic components, may underlie some of these developmentally-related learning deficits.

The expression of α1βδ GABARs is regulated by ovarian hormones. Recent reports have shown that δ expression decreases during late pregnancy (Ferando and Mody, 2013) when circulating levels of 17β-estradiol (E2) and THP are high and starting to decline, respectively. δ expression returns to pre-pregnancy levels post-partum (Ferando and Mody, 2013) suggesting a tight coupling between ovarian hormones and δ expression. Ovarian hormonal changes at puberty have a similar pattern, where circulating E2 levels increase 2-fold 5 days before the onset of puberty (Ahima et al., 1997) and THP levels rise 6–8-fold 2 weeks before the onset of puberty (Mannan and O’Shaughnessy, 1988; Fadalti et al., 1999), before declining at the time of vaginal opening (Shen et al., 2007), the physical manifestation of puberty onset. These hormonal changes likely trigger the decrease in α1βδ GABAR expression on PV+ interneurons.

In the present study, a physiological concentration of the neurosteroid THP (30 nM; Smith et al., 2006) had no significant effect on the phasic current, assessed for both inward and outward current, which is consistent with other studies showing no effect of a similar neurosteroid, THDOC (3α, 21-dihydroxy-5α-pregnan-20-one), on the phasic current below a concentration of 100 nM (Stell et al., 2003). This is in contrast to our previous findings showing a robust effect of this steroid on the tonic current generated by pubertal α4βδ GABARs. δ-containing GABARs is a sensitive target for the neurosteroids THP and THDOC (Belelli et al., 1996; Brown et al., 2002; Bianchi and Macdonald, 2003; Stell et al., 2003). However, the effect of THP at these receptors is polarity-dependent, such that 30 nM THP increases inward current (outward Cl- flux) and decreases outward current (inward Cl- flux; Shen et al., 2007; Gong and Smith, 2014) independent of Goldman rectification. This effect of the steroid was prevented by mutation of the positively charged residue arginine 353 to neutral glutamine in the intracellular transmembrane (TM)3-TM4 loop (Shen et al., 2007), suggesting this may be a re-entrant loop as has been reported for other GABARs (O’Toole and Jenkins, 2011). Neurosteroids enhance δ GABAR desensitization, which is greater for outward current (Bianchi et al., 2002) and may explain their polarity-dependent modulation of current generated by this receptor. Polarity-dependent effects are also reported for certain anesthetics (etomidate, propofol, and isoflurane) and benzodiazepines, at α1β2γ2 GABARs which have greater effects on inward current (Mellor and Randall, 1998; O’Toole and Jenkins, 2012).

In dentate gyrus granule cells where GABAergic current is depolarizing but a shunting inhibition (Staley and Mody, 1992), THDOC enhances this inhibition (Stell et al., 2003). At puberty, the GABAergic current of CA1 hippocampal pyramidal cells is outward (Shen et al., 2007). THP reduces this inhibitory tonic current at this time, increasing neuronal excitability, and increasing anxiety-like behavior (Shen et al., 2007) in contrast to its typical anxiety-reducing effect (Bitran et al., 1999).

The results from the present study suggest that synaptic GABAergic inhibition is increased at puberty in the CA1 hippocampus, both as a result of an increase in dendritic branching as well as disinhibition of the PV+ interneurons. Because tonic inhibition is also increased at this time (Shen et al., 2007), these findings suggest that the onset of puberty would represent a time of dampened hippocampal circuit activity. In humans, slower reaction times are reported for the match-to-sample task (McGivern et al., 2002) among others (Feenstra et al., 2012) in early adolescence compared with late adolescence and adulthood, in addition to sub-optimal learning. The findings from the present study may, at least in part, underlie the relative slowing down of cognitive function during early adolescence.

## Data Availability Statement

The datasets generated for this study are available on request to the corresponding author.

## Ethics Statement

The animal study was reviewed and approved by SUNY Downstate Institutional Animal Care and Use Committee.

## Author Contributions

HS conducted the electrophysiology experiments. LK conducted the immunohistochemical and Scholl analysis studies, analyzed the data, and contributed to the manuscript. SS designed the experiments, analyzed the electrophysiology experiments, made the figures, and wrote the article.

## Conflict of Interest

The authors declare that the research was conducted in the absence of any commercial or financial relationships that could be construed as a potential conflict of interest.

## Footnotes

^1^http://fiji.sc/Sholl_Analysis
